# Navigating chronic pancreatitis pain: a pathophysiological and therapeutic overview

**DOI:** 10.3389/fphys.2025.1622845

**Published:** 2025-07-10

**Authors:** Zijin Lin, Stephen Pandol, Minoti Apte, Yi Jiang

**Affiliations:** ^1^ College of osteopathic medicine, Michigan State University, East Lansing, MI, United States; ^2^ Karsh Division of Gastroenterology and Hepatology, Cedars-Sinai Medical Center, Los Angeles, CA, United States; ^3^ Pancreatic Research Group, South Western Sydney Clinical Campuses, Faculty of Medicine and Health, The University of New South Wales, Ingham Institute for Applied Medical Research, Sydney, NSW, Australia

**Keywords:** central sensitisation, chronic pancreatitis, quality of life, pain managemant, neurogenic inflammation

## Abstract

Pain management in chronic pancreatitis (CP) patients remains a major challenge, largely due to complex and refractory pain. Such pain detrimentally impacts patients by reducing quality of life, limiting daily activities, increasing psychological distress, necessitating frequent hospitalizations, and contributing to opioid dependence and socioeconomic burden. This review delineates the multifaceted nature of CP-related pain, highlighting the roles of neurogenic inflammation, maladaptive neuroplasticity, and disrupted pain modulation pathways. Current management strategies are multidisciplinary, encompassing lifestyle modification, pharmacologic therapies, endoscopic and surgical interventions, and nerve-targeted procedures (e.g., celiac plexus blocks and neurolysis). Advances in genetics, bioinformatics and biomarker research have further enhanced our understanding of CP-related pain pathogenesis, paving the way for precision medicine approaches. This review highlights current evidence and emerging innovations in the evolving landscape of CP-related pain management, emphasizing the importance of tailored and interdisciplinary care to address the intricate mechanism of CP-related pain and improve patient outcomes.

## 1 Introduction

**FIGURE 1:**
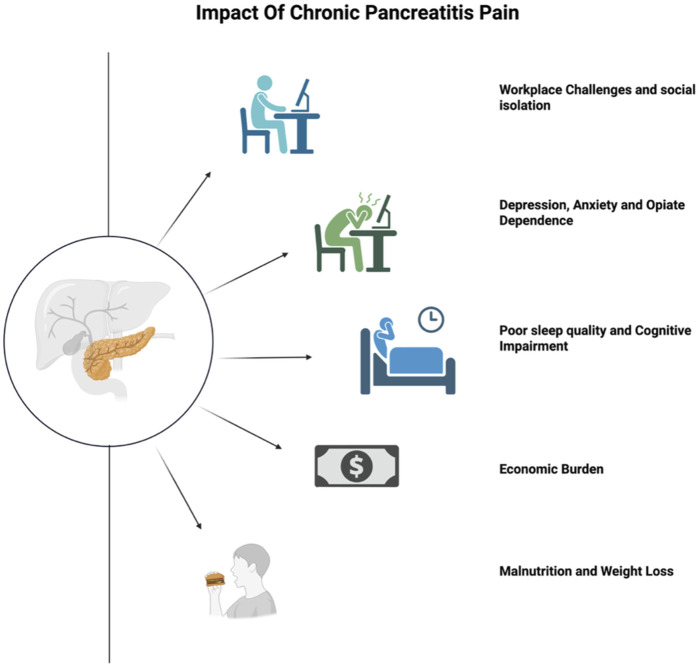
Burden of Pain from Chronic Pancreatitis. Figure created with www.biorender.com.

Chronic pancreatitis (CP) is a progressive inflammatory disorder of the pancreas characterized by a fibro-inflammatory process leading to irreversible destruction of the pancreas, ultimately resulting in fibrosis, calcification and exocrine and endocrine insufficiencies. One clinical hallmark of CP is persistent abdominal pain, commonly described as a dull, sharp or nagging sensation in the upper abdomen or radiating to the back (Yadav et al., 2023). Severe and constant pain in CP not only reduces quality of life but also impairs physical functioning, and contributes to considerable psychological distress, manifesting as anxiety, insomnia and depression (Yadav et al., 2023) (Figure 1).

CP affects an estimated 50 individuals per 100,000 in the general population (Mann et al., 2021), with risk shaped by factors such as alcohol consumption, smoking genetic predispositions, and socioeconomic status. About 85% patients report persistent pain within four to 5 years of disease onset (Ammann et al., 1984). Even after more than a decade of follow-up, up to 60% of CP patients continue experiencing painful attacks (Lankisch et al., 1995). The North American Pancreatitis Study 2 (NAPS2), the largest multi-center study of prospectively enrolled CP patients in the United States, has further revealed race-specific disparities in pain severity. Compared to white patients, Black individuals with CP exhibit more severe disease manifestations, including pronounced morphological pancreatic abnormalities, higher rates of persistent pain, increased prevalence of endocrine dysfunction, and greater disease-related disability (Wilcox et al., 2016).

CP-related pain imposes a substantial economic burden, with repeated emergency department visits, opioid prescriptions, and surgical or endoscopic interventions escalating expenses for both the healthcare system and individual patients (Wen et al., 2024).

Despite advancements in understanding CP pathophysiology, effective pain management remains a persistent challenge. Conventional treatments, including analgesics, neuromodulators, surgery, endoscopic procedures, and nerve-targeted procedures, often yield only partial relief and carry notable side effects. Additionally, the increasing reliance on opioids raises concerns about dependence and misuse. This issue is further complicated by limited data on opioid utilization in CP and the lack of standardized prescribing guidelines, leaving providers uncertain about the most effective treatment strategies (Singh et al., 2019).

## 2 Pathophysiology of pain in chronic pancreatitis

**FIGURE 2:**
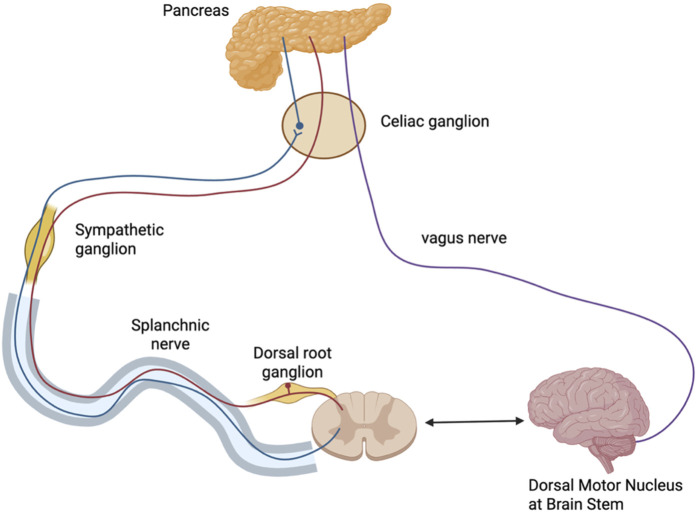
Innervation of the Pancreas. Parasympathetic fibers (purple) originate from the dorsal motor nucleus of the vagus in the brainstem and travel via the vagus nerve through the celiac region to reach intrapancreatic ganglia. Sympathetic fibers (blue) arise from the thoracic spinal cord, with preganglionic fibers projecting via the splanchnic nerve to synapse in the celiac ganglion; postganglionic fibers continue to the pancreas. Sensory afferents (red) from the pancreas travel back through the splanchnic nerve to the dorsal root ganglion and spinal cord, forming a bidirectional communication loop with the brain. Figure created with www.biorender.com.

Pain in CP is multifactorial, involving a combination of nociceptive and neuropathic components. Nociceptive pain originates from tissue inflammation and damage within the pancreas, whereas neuropathic pain is caused by injury to pancreatic or extra-pancreatic nerves, leading to disrupted nerve signaling. Prolonged inflammation also fosters structural and functional changes in nociceptive pathways, driving pain amplification and chronicity (van Zeggeren et al., 2025; Thierens et al., 2025). A foundational understanding of the normal innervation (Figure 2) of the pancreas is essential for contextualizing the mechanisms underlying pain in CP (Lkhagvasuren et al., 2021).

### 2.1 Peripheral nociception and sensitization

#### 2.1.1 Neurogenic inflammation as a key driver of peripheral sensitization

**FIGURE 3:**
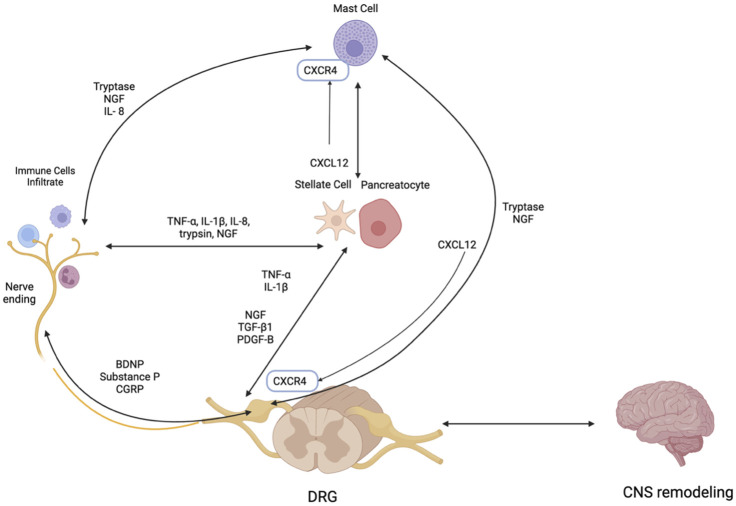
Neuroimmune Interactions in Chronic Pancreatitis: Key Mediators and Pathways. PDGF-B - Platelet-derived growth factor subunit B, CXCL 12- C-X-C motif ligand 12, CNS–Central nervous system, CXCR4 – C-X-C chemokine receptor 4, IL-1β–Interleukin-1 beta, NGF–Nerve growth factor, TGF-β1 – Transforming growth factor-beta, TNF-α–Tumor necrosis factor-alpha, NGF- Nerve Growth Factor, DRG- Dorsal Root Ganglion, BDNF–Brain-Derived Neurotrophic Factor. Figure created with www.biorender.com.

Peripheral sensitization in CP results from the heightened responsiveness of pancreatic nociceptors due to sustained exposure to inflammatory mediators. This process is driven in part by neurogenic inflammation, which is initiated by the release of neuropeptides—such as substance P, calcitonin gene-related peptide (CGRP), and brain-derived neurotrophic factor (BDNF), from sensory nerves themselves, but also other cells such as immune cells and glial cells (Figure 3).

In CP, while acinar cell injury is often the initial trigger, neurogenic inflammation can occur early and contribute to the amplification and perpetuation of the inflammatory response. Unlike inflammation initiated by immune cell infiltration, neurogenic inflammation originates from the peripheral nerve terminals themselves, through the release of neuropeptides. Experimental models of CP demonstrate that pharmacologic blockade of substance P, CGRP, and BDNF can attenuate pain (Winston et al., 2005; Fregni et al., 2007), underscoring their critical role in this process. The resulting inflammatory milieu lowers the activation threshold of peripheral sensory fibers, perpetuating pain even in the absence of ongoing pancreatic damage (Winston et al., 2005). In addition, neurogenic inflammation promotes vascular permeability, edema, and further nerve sensitization, highlighting its potential as a strategic target for therapeutic intervention (De et al., 2011).

#### 2.1.2 Critical signaling in peripheral sensitization

Peripheral sensitization is further amplified by a diverse set of mediators. Key contributors include pro-inflammatory cytokines such as tumor necrosis factor-alpha (TNF-α) and interleukin-1 beta (IL-1β), chemokines like C-X-C motif ligand 12 (CXCL12), and neurotrophic factors like nerve growth factor (NGF). These mediators alter the function of ion channels on nociceptive neurons, especially transient receptor potential vanilloid (TRPV)1 and 4 and reduce voltage-gated A-type (IA) potassium currents, lowering the threshold for pain perception. Pharmacologic antagonism of TRPV1 or TRPV4 has been shown to significantly reduce pain behaviors and hypersensitivity in CP models (Xu et al., 2007).

Pancreatic proteases such as trypsin further exacerbate sensitization by activating protease-activated receptor-2 (PAR-2) on sensory neurons, which in turn sensitizes TRPV1 (Olesen et al., 2017) and promotes the release of pro-nociceptive neuropeptides like substance P and CGRP (Olesen et al., 2017). These interactions fuel neurogenic inflammation and intensify pain signaling, especially during episodes of acute exacerbation.

NGF, normally confined to pancreatic islets, becomes widespread in CP, upregulating the TRPV1 expression and contributing to hyperalgesia (Olesen et al., 2017). In CP models, anti-NGF therapy reduces pancreatic hyperalgesia by decreasing TRPV1 current density, suppressing its expression (Olesen et al., 2017), and restoring IA potassium currents. This results in reduced nociceptor excitability and highlights NGF’s pivotal role in modulating pain pathways in CP (Zhu et al., 2011; Zhu et al., 2012a).

Additional factors such as transforming growth factor-beta1 (TGF-β1), platelet-derived growth factor B (PDGF-B), and glycoprotein 130 (GP130) are also upregulated in CP and contribute to persistent pain. TGF-β1 increases sensory neuron excitability by prolonging action potential duration, and downregulating IA currents, and its blockade has been shown to alleviate pain in CP models (Zhu et al., 2012b). Elevated levels of PDGF-B and GP130 in patients with opioid-refractory pain phenotypes in CP further implicate these molecules in sensitization and suggest a broader role for immune signaling in chronic pain maintenance (Saloman et al., 2023).

### 2.2 Peripheral neuropathy

#### 2.2.1 Pancreatic neuroplasticity and neural remodeling

In addition to neurogenic inflammation and a wide array of inflammatory and neurotrophic mediators, pancreatic neuroplasticity–characterized by increased nerve diameter and density, plays central roles in amplifying and maintaining pain in CP. This phenomenon, distinguished by intrapancreatic neural hypertrophy and hyperinnervation, is uniquely observed in CP and pancreatic cancer among pancreatic disorders (Ceyhan et al., 2009). Two key mediators driving these changes are growth-associated protein 43 (GAP-43) and neuturin. GAP-43, a critical molecule for neuronal development and axonal regeneration, is elevated in CP tissues and correlates with enhanced neural plasticity. Neurturin supports neuronal survival and differentiation. Its overexpression not only accelerates neuroplasticity but also enhances the invasiveness of pancreatic cancer cells, indicating a dual role in disease progression and pain manifestation (Wang et al., 2013; Demir et al., 2012).

Neural remodeling in CP refers to qualitative changes in pancreatic nerves, including shifts in the relative proportions of autonomic and sensory fibers (Demir et al., 2015). This process involves not only neurons but also glial cells. Notably, remodeled nerves in CP show increased expression of the neuroepithelial stem cell marker nestin and reduced expression of the glial transcription factor SOX10, suggesting enhanced activity of sprouting neural progenitor cells and potential dedifferentiation of peripheral glia. These findings point to a complex structural and cellular reorganization that serves as the qualitative basis for sustained pain and neuropathy in CP (Olesen et al., 2017; Ceyhan et al., 2010).

#### 2.2.2 Pancreatic neuritis and neuro-immune interactions

Pancreatic neuritis, the infiltration of immune cells into intrapancreatic nerves, is a hallmark feature of CP and plays a significant role in chronic pain (Demir et al., 2015). A growing body of evidence supports the contribution of neuro-immune interactions to pain mechanisms in CP, particularly through the presence of CD8^+^ cytotoxic T cells, macrophages, and mast cells (Demir et al., 2013). Among these, mast cells are especially notable for their bidirectional communication with nerve endings, a mechanism well-established in other gastrointestinal disorders such as irritable bowel syndrome (Barbara et al., 2004). This crosstalk may promote sustained neuropathic pain in CP (Buhner and Schemann, 2012).

A key chemokine signaling pathway mediating this neuro-immune interplay is the CXCL12/C-X-C chemokine receptor type 4 (CXCR4) axis. This axis is upregulated in CP and facilitates the recruitment of immune cells to perineural regions. In animal models, CXCL12 and its receptor CXCR4 are significantly elevated in the dorsal root ganglion (DRG) of CP rats, linking local immune activation to persistent pain (Zhu et al., 2017). As a result, the CXCL12/CXCR4 axis represents a potential therapeutic target, and strategies such as cytokine inhibitors, mast cell stabilizers, and anti-inflammatory agents are currently under investigation to reduce neuritis-associated pain (Cheng et al., 2019).

#### 2.2.3 Role of dorsal root ganglion in neuropathic pain

The DRG serves as a critical interface in the pathophysiology of CP-related pain, particularly in peripheral neuropathy. In CP, DRG neurons exhibit stromal hypertrophy, spontaneous firing, and axonal hyperbranching (Takamido et al., 2006), along with upregulation of pain-related ion channels and growth-associated proteins such as TRPV1 and GAP-43 (Di Sebastiano et al., 2003). These structural and molecular changes enhance DRG excitability, contributing to persistent pain signaling.

Furthermore, immune-driven inflammation, including mast cell infiltration and the release of inflammatory mediators such as IL-8 and histamine, further sensitizes DRG neurons (Demir et al., 2013). As described above, chemokine signaling via CXCL12/CXCR4 axis also plays a dual role, both in immune cell recruitment and in directly modulating DRG excitability, further bridging immune activation with peripheral neuropathic pain (Zhu et al., 2017). Altogether, the DRG acts as a dynamic modulator of neuroinflammation, making it a compelling site for therapeutic intervention in CP-associated neuropathy.

### 2.3 Central sensitization

Central sensitization is a fundamental mechanism underlying chronic pain, characterized by heightened excitability of neurons in the central nervous system (CNS) following prolonged peripheral nociceptive input (Kuhlmann et al., 2025), marked by increased synaptic efficacy, loss of inhibitory control and glial cell activation. These changes result in the amplification of pain perception (hyperalgesia) and the emergence of pain in response to non-painful stimuli (allodynia), even in the absence of ongoing tissue damage (Woolf, 2011).

#### 2.3.1 Spinal contributions to central sensitization

At the spinal level, the dorsal horn of the spinal cord plays a central role in amplifying pancreatic pain through mechanisms of central sensitization. CP induces increased spontaneous activity and excitability in DRG neurons, which represent peripheral sensitization and contribute to enhanced nociceptive input to the spinal cord. This amplified input triggers central sensitization in the dorsal horn, characterized by increased responsiveness and synaptic efficacy of second-order neurons, leading to prolonged hyperalgesia and pain independent of initial peripheral triggers (Olesen et al., 2017). Specifically, recent animal models have shown that CP induces significant upregulation of P2X7 receptor expression in spinal microglia, which mediates their activation and contributes to visceral hyperalgesia in a rat model of CP (Liu et al., 2012).

Glial cells, particularly microglia and astrocytes, play essential roles in the development and maintenance of central sensitization in CP-related pain. At the spinal cord level, microglial activation has been strongly implicated in the maintenance of visceral hyperalgesia. Liu et al. observed a marked transition of microglia from a resting to an activated state in the thoracic spinal cord 3 weeks after CP induction in rats. This prolonged activation is likely driven by persistent visceral inflammation and contributes to sustained pain states. Pharmacological inhibition of microglial activity using minocycline effectively attenuated pain behaviors, highlighting microglia as a central mediator of pain persistence in CP (Liu et al., 2012). Subsequent studies further showed that activated microglia colocalize with upregulated P2X7 receptor expression in the spinal cord, and that inhibition of P2X7 receptor significantly reduced nociceptive behaviors in CP models (Liu et al., 2012; Liu et al., 2015).

In parallel, astrocytes have also been shown to contribute to spinal central sensitization. Feng et al. demonstrated increased expression of glial fibrillary acidic protein (GFAP) in the thoracic spinal cord of CP rats, indicating astrocytic activation. Inhibition of astrocytic activation could attenuate pain of CP, suggesting that spinal astrocytes are integral to central sensitization in CP-related pain (Feng et al., 2010).

#### 2.3.2 Supraspinal structures involved in central sensitization

Supraspinally, several CNS regions have been implicated in the modulation of CP-related pain. These include the anterior cingulate cortex (ACC), anterior insular cortex (aIC), paraventricular hypothalamic nucleus (PVH), periaqueductal gray (PAG), nucleus tractus solitarius (NTS), and lateral parabrachial nucleus (LPB) (Ren et al., 2022; Bai et al., 2019a; Liu et al., 2020).

Ren et al. identified the ACC as a key cortical hub mediating both hyperalgesia and anxiety in a rat model of CP. Tract tracing and immunostaining demonstrated a direct nociceptive projection from the NTS to the ACC. CP induced glutamatergic hyperactivity in the ACC, marked by increased vesicular glutamate transporter (VGluT) 1 expression and enhanced membrane trafficking and phosphorylation of α-amino-3-hydroxy-5-methyl-4-isoxazolepropionic acid (AMPA) receptor glutamate receptor subunit 1(GluR1) and N-methyl-D-aspartate (NMDA) receptor NR2B subunits. Inhibition of ACC glutamatergic transmission through receptor antagonists or chemogenic suppression of pyramidal neurons significantly reduced pain and anxiety behaviors, highlighting the ACC as a neuromodulatory target in CP pain (Ren et al., 2022). Similarly, Bai et al. demonstrated that the aIC is critically involved in mediating both hyperalgesia and pain-related anxiety in a rat model of CP. In CP rats, aIC neurons exhibited upregulated VGluT1 expression, along with increased membrane trafficking and phosphorylation of NR2B and GluR1 subunits (Bai et al., 2019a).

The PAG has also been implicated in CP-associated central sensitization. Liu et al. found that dysfunction of the brain-gut axis in the midbrain ventrolateral PAG contributes to visceral pain, with reduced presynaptic glutamate release and diminished postsynaptic AMPA receptor-mediated responses. These neuroplastic changes weaken excitatory synaptic strength and impair descending pain inhibition (Liu et al., 2020). In a separate study, Yang et al. reported sustained activation of PAG neurons in CP models, as indicated by elevated c-Fos expression. Using a gene therapy approach to express enkephalin, they showed reduced PAG activation and visceral nociceptive behaviors, supporting a role for the PAG in mediating pain responses in CP (Yang et al., 2008).

The hypothalamus has also emerged as a relevant supraspinal region in CP pain. Recent animal studies have demonstrated that glutamatergic neurons in the PVH mediate visceral pain responses (Luo et al., 2024). Dysfunctional astrocyte-mediated glutamate clearance in this region contributes to both pain and anxiety-like behaviors in mice, implicating the PVH in affective-motivational components of CP pain (Luo et al., 2024; Ji et al., 2024).

Within the brainstem, the NTS functions as a key integration center for visceral afferents. In CP animal models, increased Fos expression, enhanced excitatory synaptic transmission, and upregulation of glutamatergic receptors (VGluT2, GluR1, NR2B) are observed within NTS (Bai et al., 2019b). Direct modulation of this region via glutamate receptor antagonists significantly reduces visceral pain, supporting the NTS as a contributor of CP-related central sensitization (Bai et al., 2019b).

Finally, the LPB has been implicated in mediating visceral hypersensitivity in CP. Wu et al. showed that CP induces glutamate release and selective NR2B upregulation in LPB neurons (Wu et al., 2025). Inhibition of NR2B receptors or LPB glutamatergic neurons significantly alleviated pain, confirming the LPB’s role in central sensitization (Wu et al., 2025).

At the supraspinal level, glial contributions have also been identified. Luo et al. reported that dysfunctional astrocytic glutamate uptake in the PVH contributes to visceral pain and anxiety in mice with CP, potentially due to deficiency of astroglial glutamate transporter-1 (GLT-1). Pharmacological activation of GLT-1 alleviated VGluT2 neuron hyperexcitability, as well as abdominal visceral pain and anxiety-like behaviors, suggesting GLT-1 as a potential therapeutic target for CP-related pain (Luo et al., 2024).

#### 2.3.3 Emerging tools and knowledge gaps in central sensitization of CP

Recent studies have begun to systematically characterize central hyperalgesia, impaired descending inhibitory pain modulation, and disrupted brain resting activity in CP patients. Notably, electroencephalogram recordings have revealed increased delta and decreased alpha peak frequencies, indicating disrupted cortical dynamics (de Vries et al., 2013). Structural and functional brain alterations have also been reported, including microstructural changes in the amygdala, cingulate cortex, and insula, as well as cortical thinning in the prefrontal and secondary somatosensory cortices. These structural changes particularly in cingulate and prefrontal cortices, identified by diffusion tensor imaging and magnetic resonance imaging (MRI), correlate with patients’ reported pain intensity (Frøkjær et al., 2011).

Despite this progress, significant gaps remain in our understanding of central sensitization in CP-related pain. For instance, the degree to which genetic predisposition, environmental exposures, or individual variability influence the development and persistence of central sensitization remains unclear. Key questions include, but are not limited to, whether certain individuals are more genetically or epigenetically predisposed to central sensitization, which risk factors contribute to the chronification of pain hypersensitivity. Additionally, improving the diagnostic accuracy of central sensitization will be essential for patient phenotyping and for selecting treatments aimed at normalizing hyperexcitable central neural circuits (Woolf, 2011). There is a critical need for further research to elucidate the precise contribution of central neural circuits to CP-related pain, which may ultimately inform more targeted neuromodulatory treatments.

### 2.4 Structural Abnormality

Traditional theories have attributed pain in CP to structural abnormalities, such as ductal obstructions, pancreatic fibrosis, and associated complications like pseudocysts. Ductal blockages elevate intraductal pressure, disrupt exocrine flow, and may induce localized ischemia, all of which can intensify nociceptive signaling via visceral nerves. Interventions aimed at reducing these obstructions (e.g., endoscopic stone removal and surgical decompression) often yield varying success in pain relief. However, studies demonstrate a limited correlation between ductal obstruction relief and long-term improvement in pain scores, suggesting that structural changes alone do not fully account for the pain experienced in CP (Whitcomb et al., 2008; Wilcox et al., 2015).

Progressive pancreatic fibrosis, a hallmark of CP, often contributes to the formation of pseudocysts. These fluid-filled structures can compress surrounding tissues and provoke severe abdominal pain. As CP advances, fibrosis and inflammation can extend to adjacent organs, including the biliary system, duodenum, stomach, and spleen, leading to complications in bile duct or duodenal strictures. Fibrotic remodeling of the duodenum results in reduced duodenal flexibility and luminal narrowing, while pseudocysts impose external pressure. While endoscopic ultrasound-guided drainage is often effective for pseudocyst-induced obstruction, surgical solutions (e.g., bypass procedures) may be necessary for fibrotic obstructions to restore gastrointestinal continuity (Bansal et al., 2019).

Recent large-center studies indicate that the severity and patterns of CP-related abdominal pain do not consistently match the extent of structural anomalies on MRI, radiographs and endoscopic imaging. Although patients with obstructive changes or with inflammatory disease more frequently report severe pain, those with pseudocysts were more likely to report mild pain (Whitcomb et al., 2008; Wilcox et al., 2015).

### 2.5 Additional pathophysiologic drivers of CP-related pain

#### 2.5.1 Genetic underpinnings of pain sensitization

Recent investigations on genetics of pain among CP patients underscore the involvement of several key pathways in peripheral sensitization, inflammation, and central sensitization. Three pancreas-related genes (CTRC, NEURL3, HSF2) have been associated with constant chronic pain, while the injury-response gene (REG3) correlates with pain severity (Dunbar et al., 2025). Further, four genes implicated in psychiatric stress disorders (TMEM65, RVFOX1, ZNF385D and LDLR) were linked to more severe pain, suggesting psychological factors exacerbate CP-related pain (Dunbar et al., 2021; Dunbar et al., 2020). Notably, four additional genes (SNYPR, NTF3, DOK6, RBFOX1) are implicated in BDNF-mediated neuropathic pain, with elevated BDNF levels observed in CP patients. These changes further lead to increased release of glutamate, CGRP, and substance P in both the periphery and CNS (Dunbar et al., 2025). Further exploration could guide the development of mechanism-based therapies, paving the way for individualized pain management in CP patients.

#### 2.5.2 Role of pancreatic stellate cells in fibro-inflammatory pain pathways

Fibrosis and persistent inflammation are central to the pathogenesis of CP and contribute significantly to associated pain. Pancreatic stellate cells (PSCs) are key mediators of this fibro-inflammatory response. In their quiescent state, PSCs store vitamin A, but become activated upon pancreatic injury or inflammation, transitioning into myofibroblast-like cells that secrete extracellular matrix proteins such as type I collagen (Masamune et al., 2009; Kong et al., 2024). Activated PSCs also release pro-inflammatory cytokines (e.g., IL-1, IL-6, TNF-α, TGF-β1), chemokines, and matrix metalloproteinases, which perpetuate immune cell recruitment and tissue remodeling. This creates a pro-inflammatory microenvironment that supports neuronal sensitization and chronic pain (Hines and Pandol, 2024). Notably, PSCs secrete neuroectodermal markers such as GFAP, as well as neurotrophins such as NGF and artemin (Jiang et al., 2020; Haas et al., 2009; Ceyhan et al., 2007); the latter could play a role in the neural hypertrophy seen in CP. Interestingly, PSCs can also activate mast cells, which in turn secrete IL-13 and tryptase, both of which activate PSCs (Ma et al., 2013). PSCs also secrete CXCL12 (Lu et al., 2022), the ligand for the axis implicated in neuropathic pain (Luo et al., 2016). Thus, PSCs are likely to play an important role in pancreatic pain, a subject that is worthy of further research. Indeed, several intracellular signaling pathways such as nuclear factor kappa-light-chain-enhancer of activated B cells (NF-κB), mitogen-activated protein kinases (MAPKs), and reactive oxygen species (ROS) that regulate PSC activation are targets for emerging therapies (Masamune et al., 2009; Masamune and Shimosegawa, 2009).

## 3 Current pain management

### 3.1 Drug therapeutics or treatment

**FIGURE 4:**
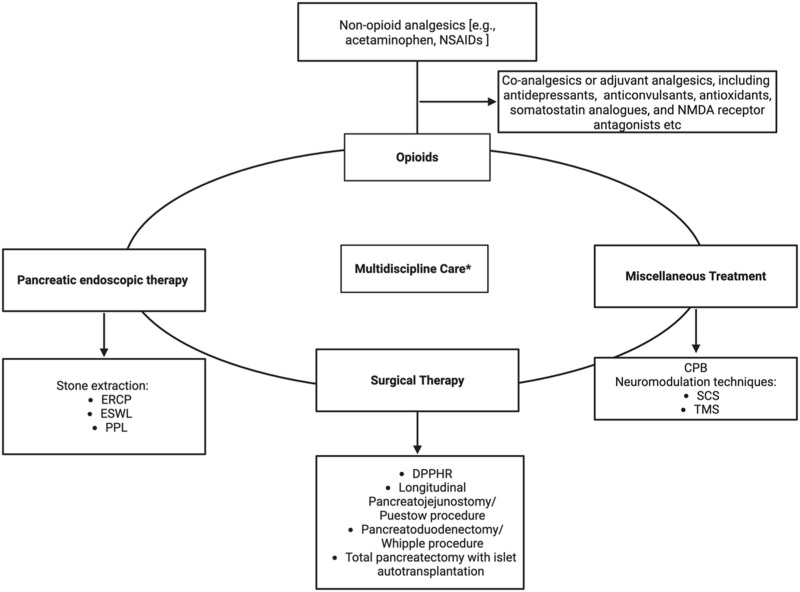
Algorithm for Multimodal Pain Management in Chronic Pancreatitis. NSAIDs–Nonsteroidal anti-inflammatory drugs, ERCP–Endoscopic Retrograde Cholangiopancreatography, ESWL–Extracorporeal Shock Wave Lithotripsy, PPL–Pancreatoscopy-guided lithotripsy, DPPHR–Duodenum-preserving pancreatic head resection, CPB–Celiac plexus block, SCS–Spinal cord stimulation, TMS–Transcranial magnetic stimulation. Figure created with www.biorender.com.

For symptomatic pain relief in CP, patients typically follow World Health Organization guidelines, starting with non-opioid analgesics [e.g., acetaminophen, nonsteroidal anti-inflammatory drugs (NSAIDs)]. Co-analgesics or adjuvant analgesics, including antidepressants and neuromodulators, are also widely employed in managing chronic visceral and neuropathic pain (Figure 4) (Table 1).

Gabapentinoids, such as pregabalin and gabapentin, have demonstrated efficacy in neuropathic pain conditions. Recent animal studies suggest that gabapentin may potentiate the analgesic effect of morphine in pancreatitis (Smiley et al., 2004). Antidepressants, including serotonin-norepinephrine reuptake inhibitors (SNRIs) and tricyclic antidepressants (TCAs), are frequently used as neuromodulators. While direct evidence in CP remains limited (Lunn et al., 2014), most authoritative guidelines acknowledge their potential role (Talukdar and Reddy, 2013). However, recommendations are largely based on lower-level evidence and extrapolation from other chronic neuropathic pain conditions.

Oxidative stress plays a significant role in the pancreatic injury and contributes to persistent inflammation. Both clinical and experimental studies have demonstrated increased production of ROS and depletion of endogenous antioxidants, particularly within pancreatic acinar and stellate cells. Accordingly, antioxidant therapy has been proposed as a disease-modifying intervention. A combination of micronutrients—commonly including methionine, selenium, vitamins C and E, and β-carotene—has been shown to restore systemic antioxidant capacity (Zhou et al., 2015). Clinical trials and meta-analyses suggest that such supplementation may reduce pain intensity and analgesic consumption in a subset of patients, presumably through suppression of ROS-mediated inflammatory signaling and modulation of neuroimmune interactions within the pancreas (Talukdar et al., 2015; Zhou et al., 2015). However, the precise mechanisms by which oxidative stress influences nociceptive processing in CP remain to be fully elucidated.

Often, managing pain in CP necessitates the use of opioid therapy. Tramadol, a weak opioid agonist that inhibits norepinephrine and serotonin reuptake, is frequently utilized for moderate pain management in CP due to its more favorable side-effect profile compared with stronger opioids like morphine (Edinoff et al., 2021). For more severe or persistent pain, stronger opioid, such as fentanyl patches, may be considered particularly for patients who have difficulty swallowing or adhering to oral medication regimens (Fu et al., 2024). However, achieving adequate analgesia often necessitates doses above the manufacturer’s recommendations. Thus, these agents should be reserved for individuals with established opioid tolerance or dysphasia (Niemann et al., 2000).

Less commonly used agents also show promise in treating refractory pain. Somatostatin analogues and ketamine have been explored for pain relief (Horváth et al., 2022), although their clinical benefits remain primarily anecdotal. Additionally, clonidine, benzodiazepines, antipsychotics and cannabinoids have been used off-label for refractory CP pain clinically.

In cases where analgesic therapy fails to adequately control pain, current practice emphasizes a multimodal approach tailored to the patient’s specific anatomy, disease etiology, and symptom severity, rather than strictly adhering to a linear progression of medical to surgical therapies. Previously, it was thought that in cases where pain persists despite prolonged use of analgesics (often opioids) and multiple endoscopic procedures, up to 75% of patients may ultimately require invasive surgical intervention (Bouwense et al., 2015).

### 3.2 Endoscopic intervention

Pancreatic endoscopic therapy is most frequently indicated for CP patients with proximal main pancreatic duct obstructions, commonly a single stone or a stricture in the head of the pancreas (Strand et al., 2022). Early intervention tends to yield better outcomes, before extensive fibrotic or inflammatory changes become entrenched.

Two primary techniques that address pancreatic duct obstruction are stenting and stone extraction. Stenting involves the placement of pancreatic duct stents to relieve obstruction and facilitate ductal drainage, while stone extraction involves endoscopic balloon retrieval and often combined with extracorporeal shockwave lithotripsy (ESWL) to fragment and remove intraductal stones (Tandan et al., 2019).

In cases of a dominant stricture in the main pancreatic duct that results in significant ductal dilation, ductal stenting is often employed alongside endoscopic therapy. Stents help to bypass the obstruction, restore ductal flow, and alleviate the buildup of intrapancreatic pressure that contributes to pain and tissue damage.

Most recent guidelines suggest that small (≤5 mm) stones should be treated with pancreatography and conventional stone extraction maneuvers under Endoscopic Retrograde Cholangiopancreatography (ERCP) (Strand et al., 2022). For patients with large obstructive stones (>5 mm) in the pancreatic head, endoscopic therapy is frequently combined with ESWL (Tandan et al., 2019). ESWL uses focused acoustic waves to fragment pancreatic duct stones into smaller pieces, allowing their subsequent removal through endoscopic techniques (Drewes et al., 2017). Evidence supports the efficacy of ESWL; short-term pain relief is reported in around 85% of patients receiving endotherapy, with long-term improvements observed in around two-thirds of patients (Talukdar et al., 2024). Pancreatoscopy-guided lithotripsy, a newer technology, is also commonly employed and has achieved 88%–100% stone clearance compared to ∼68% with ESWL (Gerges et al., 2023; van Huijgevoort et al., 2020).

The success and timing of pancreatic endoscopic therapy in CP are influenced by a complex interplay of factors, including patient demographics, disease progression, and socioeconomic status. For example, women and individuals with lower income are more likely to receive endoscopic treatment (Machicado et al., 2018). One study noted that endoscopic therapy typically occurred within 1 year after CP diagnosis, involved a median 2-month treatment span, and an average of two sessions per course (Issa et al., 2020; Clarke et al., 2012). Although ∼65% of patients experience improvement, recurrent ductal strictures or stones lead 30%–50% to need additional procedures within 2–3 years (Tringali et al., 2019; Han et al., 2024). Roughly one-third showed no response to their initial endoscopic therapy course (Issa et al., 2020), and only a small subset of patients proceeded to surgery, despite randomized controlled trial evidence suggesting that early surgical intervention may offer superior pain relief (Issa et al., 2020; Díte et al., 2003; Bordaçahar et al., 2018).

### 3.3 Surgical management

Surgical intervention for CP plays a critical role in disease management, with its timing and sequence relative to medical and endoscopic therapies remaining a subject of ongoing debate. While traditionally reserved for patients with severe, long-standing symptoms refractory to conservative treatments, emerging evidence suggests potential benefits of earlier surgical intervention in select cases.

Pancreatoduodenectomy (Whipple procedure) is typically indicated for disease predominantly affecting the pancreatic head, this involves the removal of the pancreatic head along with the duodenum and other adjacent structures. It effectively relieves pain but carries a substantial risk of loss of pancreatic function (Andersen and Frey, 2010). Duodenum-preserving pancreatic head resection (DPPHR) including procedures such as Frey and Beger/Berne techniques, targeting the inflamed pancreatic head while preserving the duodenum and maintaining pancreatic function. Comparative studies have shown that these procedures provide pain relief equivalent to pancreatoduodenectomy, with superior preservation of exocrine and endocrine functions. A meta-analysis comparing pancreatoduodenectomy and DPPHR suggests that both are equally effective in improving quality of life and pain relief; however, DPPHR is generally preferred due to its lower complication rates and better functional outcomes (Guo et al., 2023).

Longitudinal pancreatojejunostomy (Puestow procedure) involves creating a drainage channel by opening the pancreatic duct along its length and attaching it to the jejunum. This approach is particularly useful for patients with a diffusely dilated duct and widespread disease (Tchouta and Schrope, 2024). Distal pancreatectomy is indicated for disease localized to the body or tail of the pancreas. While this procedure can effectively provide pain relief, it may result in loss of pancreatic endocrine and exocrine functions (Hallac et al., 2020).

For patients with intractable pain who have exhausted other treatment modalities, total pancreatectomy with islet autotransplantation (TPIAT) is an advanced option (McEachron and Bellin, 2018; Bellin et al., 2017a). For children with genetic risk factors or greater disease burden, it may be utilized earlier (Bellin et al., 2017b; Heinzman et al., 2023). This procedure entails the complete removal of the pancreas, followed by transplantation of isolated islets into the liver to preserve insulin production. A meta-analysis conducted by the Dutch Pancreatitis Study Group has shown an increase in opioid-free rates from 0% to 15% pre-operatively to 63% postoperatively, with insulin dependence rate decreasing to 30% (Kempeneers et al., 2019). Observational data from Sutherland et al. showed durable pain relief in 85% of patients, with 60% achieving narcotic independence and approximately 30% attaining insulin independence (Sutherland et al., 2012). Additionally, a prospective study also reported reduced pain severity in young children with severe CP (Heinzman et al., 2023).

The optimal sequence of using endoscopic procedure or surgery to relieve pain in CP patients remains a topic of debate among clinicians. Cahen et al. demonstrated that early surgery provided better pain control over 24-month, whereas endoscopic treatment required more procedures (Cahen et al., 2007). Similarly, the ESCAPE trial showed a significant lower integrated pain score in the early surgery group compared with endoscopy-first group (37 vs. 49) at 18-month follow up (Issa et al., 2020). A subsequent follow-up publication confirmed that the early surgery group had superior outcomes compared to the endoscopy-first group, demonstrating better pain control (as measured by Izbicki and VAS pain scores), higher rates of complete pain relief, and greater patient satisfaction (van Veldhuisen et al., 2025). Despite concerns over strict inclusion criteria and limited generalizability, findings from ESCAPE trial and the follow-up data challenge the traditional paradigm of reserving surgery as a last resort. Instead, they suggest that earlier surgical interventions may provide significant benefits for select patients.

### 3.4 Miscellaneous treatment

Celiac plexus block (CPB) is a standard intervention for managing pain associated with CP and other upper abdominal conditions. Steroid injections, most commonly triamcinolone, provide temporary relief by reducing local inflammation and edema (Cornman-Homonoff et al., 2017; Sheth et al., 2024). CPB alleviates CP-related pain by targeting the celiac plexus, disrupting nociceptive pathways and reducing peripheral input to the CNS. This may mitigate central sensitization and, through autonomic modulation, relieve ischemia and inflammation-related discomfort (Shienbaum, 2024).

Traditionally performed via percutaneous techniques under fluoroscopic or computed tomography (CT) guidance, CPBs increasingly utilize endoscopic ultrasonography (EUS), which offers advanced imaging visualization of the celiac axis and surrounding structures through Doppler imaging. In one trial, CPB reduced pain scores in 70% of patients with CP (Santosh et al., 2009). This approach is particularly beneficial in managing CP-related pain, where pain is often driven by local inflammation secondary to the release of pancreatic enzymes (Wilcox et al., 2024; Wyse and Sahai, 2018).

For more enduring relief, neurolytic agents such as ethanol and phenol are employed to chemically ablate the nerve fibers of the celiac plexus (Hooten, 2024). Neurolytic interventions are primarily reserved for malignant conditions or refractory CP pain due to their irreversible effects (Noble and Gress, 2006). A currently ongoing clinical trial is evaluating the feasibility of EUS-guided CPB and neurolysis (Wilcox et al., 2024). Common side effects of CPB include transient hypotension, diarrhea, and localized back pain (Dos Santos Silva et al., 2023). Neurolytic procedures, due to their more destructive nature, may result in rare (<2%) (Kambadakone et al., 2011), but more serious complications such as orthostatic hypotension, retroperitoneal hemorrhage, or unintended motor and sensory deficits secondary to inadvertent neural injury (Shienbaum, 2024). Therefore, careful patient selection and procedural planning are essential.

Neuromodulation techniques, such as spinal cord stimulation (SCS) and transcranial magnetic stimulation (TMS), are emerging as potential adjuncts for managing refractory pain in CP. SCS delivers electrical stimulation to the dorsal columns, activating large-diameter Aβ fibers that suppress nociceptive input from smaller fibers. This reduces pain perception via mechanisms aligned with gate control theory and enhancement of descending inhibitory pathways (Deer et al., 2014). SCS involves the implantation of electrodes near the spinal cord, usually T6-T8, to deliver electrical impulses, achieving pain relief in approximately 50%–60% of patients, with a 6% infection rate as the primary complication (Ratnayake et al., 2020).

TMS, a non-invasive neuromodulation technique using magnetic fields to stimulate specific brain regions (typically the primary motor cortex or dorsolateral prefrontal cortex), has shown promise in neuropathic pain and depression, but remain under investigation for CP-related pain (Yang and Chang, 2020). Preliminary results suggest feasibility and safety of TMS, though larger, disease-specific trials are needed (Yang and Chang, 2020; O'Connell et al., 2018). Its analgesic effects are thought to involve modulation of cortical excitability and enhancement of descending pain inhibition, likely mediated by changes in thalamic and limbic activity as well as alterations in neurotransmitters (Lefaucheur et al., 2020).

## 4 Emerging diagnostics and therapeutic approaches

### 4.1 Pain phenotyping: the role of pancreatic-specific quantitative sensory testing in CP-related pain

Phenotyping of pain in CP is an emerging area of research aimed at improving diagnostic accuracy and personalizing treatment. Quantitative Sensory Testing (QST), particularly the pancreatic-specific QST (P-QST), has showed potential in differentiating pain subtypes and identifying central sensitization. By applying controlled stimuli, P-QST helps elucidate underlying pain mechanisms, with a specific emphasis on segmental and widespread hyperalgesia as indicators of central sensitization. This approach is especially valuable given that central sensitization, a key driver of pain chronicity in CP, is often challenging to detect using conventional imaging or routine clinical assessments (Buscaglia and Chang, 2022; Faghih et al., 2022).

An ongoing observational clinical trial is evaluating the predictive value of P-QST phenotyping for pain relief following endoscopic or surgery interventions in 150 CP patients recruited from three U.S. tertiary care centers. Participants are classified as having no central sensitization, segmental sensitization, or widespread hyperalgesia prior to treatment. Six-month pain reduction via a Numeric Rating Scale, serves as the primary outcome. These findings are expected to guide the development of individualized treatment strategies based on P-QST results (Phillips et al., 2024).

### 4.2 Emerging therapies and ongoing clinical trials for CP and associated pain

A growing body of research highlights the importance of novel interventions that target not only the pancreatic pathology but also the multifaceted nature of CP-related pain. These emerging therapies encompass novel pharmacologic agents, minimally invasive procedural innovations, and digital health solutions, all of which aim to alleviate pain while addressing key aspects of CP pathophysiology (Table 2).

**TABLE 1:** Non-opioid pharmacologic interventions for chronic Pancreatitis pain: Mechanisms, evidence, and side effects.

Drug category	Example	Mechanism of action	Key evidence	Potential side effects
Gabapentinoids	Gabapentin, Pregabalin	- Bind to the α2-δ subunit of presynaptic, voltage-gated calcium channels, reducing calcium influx and decreasing the release of excitatory neurotransmitters (Rusciano, 2024), thereby decreasing neuronal excitability and alleviating neuropathic pain - Pregabalin has a six-fold higher binding affinity than gabapentin, enhancing potency (Mayoral et al., 2024) - Pregabalin exhibits linear pharmacokinetics (Fink et al., 2002), leading to improved adherence and consistent drug levels (Chincholkar, 2020)	- RCTs show pregabalin significantly reduces pain intensity in patients with refractory CP pain, especially post-surgical or post-endoscopic intervention (Olesen et al., 2011; Bouwense et al., 2012) - Combination therapy with pregabalin and antioxidants showed superior pain reduction compared to placebo for treating CP related pain (Bouwense et al., 2012)	- Dizziness, somnolence, peripheral edema, weight gain
SNRIs	Duloxetine, Venlafaxine	- Increase levels of serotonin and norepinephrine, thereby enhancing descending inhibitory pain pathways and reducing nociceptive transmission - Potentially attenuate central sensitization by modulating the release of excitatory neurotransmitters (Temmermand et al., 2022)	- Current evidence is limited to smaller studies or extrapolations from other chronic pain conditions. There is no well-established, high-level RCT evidence specifically validating SNRIs for CP pain, so their use is primarily guided by clinical judgment and the broader chronic pain literature (Lunn et al., 2014)	- Nausea, headache, dizziness, elevated blood pressure - Drug interactions for serotonin syndrome (e.g., Triptans, Linezolid, MAOIs) (Francescangeli et al., 2019)
TCAs	Amitriptyline, Nortriptyline, Desipramine	- Inhibit the reuptake of norepinephrine and serotonin, enhancing descending inhibitory pathways (Moraczewski and Aedma, 2023) - Peripheral activation of α_2_-adrenergic receptors on nerve terminals, reducing neurotransmitter release (Pertovaara, 2006)	- No strong evidence from large, multi-center RCTs, but small pilot trials and clinical experience support their cautious use, especially for neuropathic or centralized pain mechanisms. Recommendations based on extrapolation from other pain states (Drewes et al., 2017)	- Anticholinergic effects (dry mouth, sedation, constipation, urinary retention), QT prolongation, orthostatic hypotension (Moraczewski and Aedma, 2023)
Antioxidants	methionine, organic selenium, β-carotene, ascorbic acid, and α-tocoferol (Talukdar et al., 2016)	- Reduce oxidative stress and inflammation in pancreatic tissue, mitigating pain (Swentek et al., 2021)	- Meta-analyses show reduction in CP related pain intensity and frequency with combined antioxidant therapy (Talukdar et al., 2015) - Combination with pregabalin showed additional benefit in one RCT for CP (Talukdar et al., 2016)	- Gastrointestinal upset, nausea, and variability in formulation consistency
Somatostatin Analogues	Octreotide	- Inhibit pancreatic secretions, reducing ductal pressure and inflammation (Horváth et al., 2022)	- Limited evidence for CP but some efficacy demonstrated in acute pancreatitis	- Nausea, diarrhea, gallstones, injection site pain
NMDA Receptor Antagonists	Ketamine	- Block NMDA receptors in the CNS, reducing central sensitization and modulating pain perception (Ketamine, 2023)	- Anecdotal evidence for refractory CP pain; used off-label in clinical practice	- Psychotropic side effects (hallucinations, dysphoria), hypertension, nausea
Others	Clonidine	- Alpha-2 adrenergic agonist; reduce sympathetic outflow, inhibit nociceptive neurotransmission, and modulate pain pathways (Amna et al., 2024)	- Occasionally used off-label for CP pain; formal evidence limited	- Hypotension, bradycardia, sedation, dry mouth
	Cannabinoids	- Interact with CB1 and CB2 receptors, modulating pain perception and inflammation (Goodman et al., 2025)	- Limited evidence, but potential role in refractory pain management; ongoing trials are exploring efficacy (Johnson et al., 2025)	- Drowsiness, dizziness, dry mouth, legal and regulatory considerations

SNRI, Serotonin-Norepinephrine Reuptake Inhibitor; CNS, Central Nervous System; RCT, Randomized Controlled Trial; TCA, Tricyclic Antidepressant, NMDA–N-Methyl-D-Aspartate, CB1 – Cannabinoid Receptor Type 1, CB2 – Cannabinoid Receptor Type 2, CP, Chronic Pancreatitis; MAOIs, Monoamine Oxidase Inhibitors.

**TABLE 2:** Ongoing clinical trials exploring emerging therapies for chronic pancreatitis pain management.

NCT Number	Title	Enrollment	Study type	Intervention	Locations	Estimated completion
NCT06426160	Tocilizumab for Painful Chronic Pancreatitis	36	A randomized, placebo-controlled trial evaluating tocilizumab for reducing CP pain by reducing inflammation and fibrosis	Drug: Tocilizumab, Sodium Chloride	Europe	2026-6
NCT04115826	ESWL vs. PPL for Painful Chronic Calcific Pancreatitis	150	A randomized trial comparing ESWL and PPL for pancreatic duct stone clearance and pain relief	Procedure: ESWL, PPL	US	2026-6
NCT041572	Role of Home-Based TEA for Treatment of Pain in Chronic Pancreatitis	40	A crossover trial assessing TEA, a non-invasive therapy stimulating acupuncture points via electrical currents to modulate pain pathways	Device: TEA	US	2027-12
NCT063262616	QOLAPI: Quality of Life Assessment of Chronic Pancreatitis Endoscopic Interventions	500	A multicenter cohort study evaluating endoscopic approaches to relieve ductal obstruction and inflammation	Endoscopic Procedures	US	2026-8
NCT06362187	VR Pilot for Pancreatitis	20	A pilot study exploring VR integrating cognitive-behavioral techniques to improve pain perception and emotional wellbeing	Device: Gut-Directed VR, Sham Control	US	2026-5
NCT06178315	EUS-CPB vs. Sham in Chronic Pancreatitis	94	A randomized trial investigating EUS-CPB, using steroids and anesthetics to inhibit sympathetic nerve activity and relieve CP pain	Procedure: EUS-Guided Celiac Plexus Block	US	2026-12
NCT04043074	EUS-CPN for Chronic Pancreatitis	35	A prospective trial examining EUS-CPN, where neurolytic agents disrupt sympathetic pathways for long-term pain relief	Procedure: Celiac Plexus Neurolysis	US	2029-10
NCT05925036	Novel Cellular Therapy for the Treatment of Pain Associated with Chronic Pancreatitis	40	A phase 1 trial assessing Mesenchymal stromal cell for their anti-inflammatory and immunomodulatory properties in reducing CP-related fibrosis, inflammation, and pain	Drug: Mesenchymal Stem Cells, Placebo	US	2027-12
NCT05603702	STTEPP: Safety, Tolerability, and Dose-Limiting Toxicity of Lacosamide in Patients with CP Pain	24	A dose-escalation trial testing lacosamide as an adjunct to opioids to reduce hyperalgesia and central sensitization	Drug: Lacosamide	US	2025-3
NCT05664880	ALLIANCE: A Pilot Clinical Trial of Paricalcitol for Chronic Pancreatitis	24	A pilot study evaluating paricalcitol for its anti-inflammatory and antifibrotic effects in CP pain management	Drug: Paricalcitol	US	2026-1
NCT05771675	Simvastatin Treatment to Improve Patient-Reported Outcomes in Patients with CP	90	A double-blind trial testing potential effect from simvastatin to enhance autophagic clearance and reduce fibro-inflammatory responses to improve CP pain outcomes	Drug: Simvastatin, Placebo	US	2026-6

ESWL- extracorporeal shock wave lithotripsy, PPL, Pancreatoscopy-Guided Lithotripsy; TEA, Transcutaneous Electrical Acustimulation; VR, Virtual reality; EUS-CPB, endoscopic ultrasound-guided celiac plexus block; EUS-CPN, Endoscopic Ultrasound-Guided Celiac Plexus Neurolysis.

Among pharmacologic therapies, tocilizumab, an anti-IL-6 monoclonal antibody, is under investigation in the TOPAC trial (Hagn-Meincke et al., 2025), for its potential to reduce systemic and pancreatic inflammation, thereby potentially normalizing pain processing and improving quality of life. IL-6 has emerged as a key mediator in the complex immune signaling networks of CP, based on evidence from both preclinical models and human studies using pancreatic cell lines and tissue samples (Komar et al., 2017). In peripheral sensitization and pancreatic neuroplasticity models, IL-6 appears to be upregulated in both the pancreas and thoracic DRG, sensitizing primary afferent nociceptors via autocrine or paracrine mechanisms, including enhanced TRPV1 activity (Xu et al., 2007). Clinically, a recent pilot study showed a progressive rise in plasma IL-6 levels correlating with the severity of CP, suggesting its utility as a biomarker across disease stages (Hagn-Meincke et al., 2024). By blocking both membrane-bound and soluble forms of IL-6 receptor (IL-6R), tocilizumab disrupts downstream JAK/STAT and MAPK signaling, attenuating inflammatory and fibrotic cascades as well as nociceptive transmission (Chen et al., 2023). While prior studies have shown its effectiveness in modulating systemic inflammation in acute pancreatitis (Chen et al., 2016), emerging evidence supports its potential in chronic, immune-driven pain phenotypes in CP (Chen et al., 2023). The novel small molecule IL-6R antagonist TB-2–081 has also demonstrated effectiveness in reversing CP-induced referred abdominal hypersensitivity in experimental models (Vardanyan et al., 2010).

Lacosamide, a sodium channel blocker, has garnered interest for its selective modulation of NaV1.7—a voltage-gated sodium channel critical in amplifying subthreshold depolarizations and increasing the excitability of nociceptive neurons, particularly within the DRG and peripheral sensory pathways. By enhancing the slow inactivation of NaV1.7, lacosamide reduces neuronal hyperexcitability associated with chronic pain (Dib-Hajj et al., 2013). This mechanism positions it as a potential therapeutic candidate for managing pain in CP (Fogel et al., 2024). In addition, it is currently under evaluation to determine its ability to mitigate opioid-induced hyperalgesia, a frequent complication in patients with CP who rely on long-term opioid therapy (Fogel et al., 2024).

Emerging evidence also supports the role of simvastatin, traditionally used as a lipid-lowering agent, in restoring acinar cell homeostasis and modulating fibro-inflammatory pathways, which are critical in CP pathogenesis. Mechanistically, simvastatin and other HMG-CoA reductase inhibitors suppress PSC activation by inhibiting the mevalonate pathway, which disrupts isoprenylation and membrane localization of Ras and Rho GTPases. This downregulates the Ras-Raf-ERK pathway, inhibits PDGF-induced RhoA activation, reduces α-smooth muscle actin expression, and promotes apoptosis in activated PSCs, thereby attenuating fibrogenic signaling (Jaster et al., 2003).

Paricalcitol, a vitamin D analog, is another agent under study for its dual anti-fibrotic and anti-inflammatory properties. It acts by activating the vitamin D receptor on PSCs, promoting transcriptional reprogramming that suppresses PSC activation and extracellular matrix production. Mechanistically, this includes inhibition of TGF-β/SMAD signaling, downregulation of pro-fibrotic genes, and a shift of PSCs toward a quiescent phenotype, resulting in reduced inflammation and stromal fibrosis (Sherman et al., 2014).

Early data also suggest that mesenchymal stem cell transplantation may exert regenerative properties on the inflamed pancreas, potentially reducing fibrosis and pain by suppressing inflammatory cytokines and PSCs (Kawakubo et al., 2018).

Procedural innovations remain a cornerstone of pain management in CP. Endoscopic approaches, such as EUS-CPB and EUS-guided celiac plexus neurolysis (EUS-CPN), represent minimally invasive methods to achieve localized nerve inhibition. Ongoing trials are assessing their safety and efficacy in CP patients with refractory pain. Lithotripsy Techniques such as ESWL and pancreatoscopy-guided lithotripsy are both under evaluation to determine the most effective approach for achieving pancreatic duct stone clearance. Successful duct clearance can significantly reduce pain and mitigate complications related to ductal hypertension.

Incorporating digital health tools and behavioral interventions is also a growing area of focus in managing CP-related pain. Among these, cognitive behavioral therapy reduces pain by modulating central sensitization (Wang et al., 2025). The Internet-Delivered Pain Self-Management for Persons With Acute Recurrent and Chronic Pancreatitis Pain (IMPACT-2) trial explores an internet-delivered pain self-management program that teaches essential skills like relaxation, goal-setting, and activity pacing. These skills empower patients to actively participate in their pain management, potentially improving both adherence and outcomes. Virtual reality therapy aims to reshape pain perception by leveraging immersive technology, engaging cognitive-behavioral techniques, and altering attitudes, emotions, and coping mechanisms related to pain.

Transcutaneous electrical acustimulation (TEA), provides a non-invasive, home-based option to modulate pain pathways without needles or medication, thus broadening the range of pain management strategies. TEA has demonstrated analgesic effects in patients with early-stage acute pancreatitis, primarily through autonomic modulation (decreasing sympathetic tone while enhancing vagal activity) to reduce visceral hypersensitivity and improve gastrointestinal motility (Xuan et al., 2022). Its role in CP-related pain, however, remains to be clarified.

## 5 Conclusion

Advancements in CP pain management will hinge on enhanced mechanistic understanding, precision diagnostics, and targeted therapies. Future research aims to integrate large cohort studies and big data to identify genetic markers and refine risk stratification, while artificial intelligence (AI) and advanced imaging techniques will enable more precise diagnostics and prediction of disease progression. Mechanistic studies focusing on inflammation, fibrosis, and neuropathy may uncover genetic pathways for tailored treatments. Novel and repurposed therapies, including paricalcitol, statins, lacosamide, and cystic fibrosis transmembrane conductance regulator (CFTR) modulators, etc., are generating optimism that they may address CP’s underlying pathophysiology. Additionally, digital health tools, such as AI-assisted cognitive behavioral therapy and wearable sensors, provide new non-pharmacologic and objective outcome measures. Optimizing clinical trial designs by incorporating genetic profiles, tailoring interventions, and quantifying placebo effects will improve therapeutic efficacy in this heterogeneous disease. These measures aim to refine treatment approaches, ultimately improving patient outcomes and quality of life.
